# Spectrophotometric Determination of *N*-Acetyl-L-Cysteine and *N*-(2-Mercaptopropionyl)-Glycine in Pharmaceutical Preparations

**DOI:** 10.1155/2011/140756

**Published:** 2011-05-23

**Authors:** Lea Kukoc-Modun, Njegomir Radić

**Affiliations:** Department of Analytical Chemistry, Faculty of Chemistry and Technology, University of Split, Teslina 10/V, 21000 Split, Croatia

## Abstract

A simple spectrophotometric method for the determination of *N*-acetyl-L-cysteine (NAC) and *N*-(2-mercaptopropionyl)glycine (MPG) in pharmaceutical preparations was developed, validated, and used. The proposed equilibrium method is based on a coupled two-step redox and complexation reaction. In the first step, Fe(III) is reduced to Fe(II) by NAC or MPG. Subsequently, Fe(II) is complexed with 2,4,6-tripyridyl-s-triazine (TPTZ). Several analytical parameters of the method were optimized for NAC and MPG analysis in the concentration range from 1.0 *μ*M to 100.0 *μ*M. Regression analysis of the calibration data showed a good correlation coefficient (0.9999). The detection limit of the method was 0.14 *μ*M for NAC and 0.13 *μ*M for MPG. The method was successfully applied to quantify NAC and MPG in pharmaceutical preparations. No interferences were observed from common pharmaceutical excipients.

## 1. Introduction

*N*-Acetyl-L-cysteine (NAC) is an endogenous aminothiol present both in human plasma and in urine [1]. *N*-(2-Mercaptopropionyl)glycine (MPG), also known as tiopronin, is a synthetic aminothiol antioxidant. NAC has been in clinical use for more than 40 years, primarily as a mucolytic agent in a variety of respiratory illness. Intravenous and oral administration of NAC have been extensively used in the management of paracetamol (acetaminophen) poisoning [1]. MPG is primarily used in the treatment of cystinuria [2], but studies have shown that MPG can be used as a chelating, cardioprotecting, and radioprotecting agent [3], as well as an antidote to heavy metal poisoning [4].

A number of electrochemical [5–9], fluorometric [10–12], chemiluminescence [13–15], and liquid chromatographic [16–18] methods have been developed for the determination of NAC and MPG in biological samples and pharmaceuticals. Some of these methods are in part time consuming or require expensive equipment. Other published methods suffer from lack of selectivity and sensitivity. Spectrophotometry is the most widely used technique in pharmaceutical analysis because it is simple, economic, and easily available to most quality control laboratories. Spectrophotometric methods have also been reported for the determination of NAC and MPG in pharmaceutical formulations [19–25]. 

A coupled redox-complexation reaction has been reported for the spectrophotometric analysis of NAC and MPG using 1,10-phenanthroline as the chromogenic reagent [24]. In the present work, we report a simple and cost-effective spectrophotometric method for the reliable analysis of NAC and MPG in pharmaceutical formulations. The method is also based on the coupled redox-complexation reaction between NAC or MPG and Fe(III) but uses 2,4,6-tripyridyl-s-triazine (TPTZ) as the chromogenic reagent. Collins et al. have introduced TPTZ as chromogenic reagent for determination of Fe(II) [26]. The Fe(II) complex with TPTZ has a twice higher molar absorptivity coefficient (2.2 × 10^4^ L mol^−1^ cm^−1^) than the Fe(II) complex with 1,10-phenanthroline (1.1 × 10^4^ L mol^−1^ cm^−1^). TPTZ provides higher selectivity, linearity, and sensitivity of the method.

## 2. Material and Methods

### 2.1. Materials

All spectrophotometric studies were carried out on an ultraviolet-visible, double-beam spectrophotometer (UV-1601 SHIMADZU, Kyoto, Japan), and using 1 cm quartz cells. The spectrophotometer was coupled to a personal computer. Measurements of pH were carried out with a Mettler Toledo SevenMulti potentiometer (Mettler Toledo, Schwerzenbach, Switzerland) equipped with a combined glass electrode Mettler Toledo In Lab 413. A thermostated water bath (MGW Lauda, Germany) was used to keep a constant cuvette temperature of 25 ± 0.5°C.

### 2.2. Chemicals and Reagents

All chemicals were of analytical-reagent grade, and solutions were prepared in MilliQ deionised water. All stock solutions were stored at 4°C in dark bottles. Separate 10 mM stock solutions of NAC and MPG were prepared by dissolving 163.2 mg (1 mmol) of NAC (Merck, Darmstadt, Germany) or 163.2 mg (1 mmol) of MPG (Sigma-Aldrich, St. Louis, USA) in deionised water up to 100.0 mL volume and stored in the dark bottle at 4°C. Dilutions were prepared daily in deionised water. 

Stock solution of Fe(III) (10 mM) was prepared by dissolving 270.3 mg (1 mmol) of FeCl_3_ × 6 H_2_O (Kemika, Zagreb, Croatia) in 50.0 mL deionised water. Then 0.5 mL of concentrated HCl was added and the volume was adjusted to 100.0 mL with deionised water.

A stock solution of 10 mM TPTZ (Merck, Darmstadt, Germany) was prepared by dissolving 312.3 mg (1 mmol) TPTZ in 2.0 mL of a 6.0 M HCl, followed by addition of deionised water up to a total volume of 100.0 mL. 

Acetate buffer (0.5 M) was used to cover the pH range 3.2–4.0. For solutions of pH 1.0 and 2.0, 0.1 M HCl and 0.01 M HCl were used, respectively.

Two different pharmaceutical formulations of NAC were analysed by the present spectrophotometric method, that is, Fluimukan 200 mg granules, and Fluimukan Akut 600 mg dispersible tablets (Lek, Ljubljana, Slovenia). The content of five granules was powdered by means of a mortar. An accurately weighed portion of the powder containing about 200 mg of NAC was transferred into a 500 mL volumetric flask, and NAC was dissolved in and diluted to the nominal volume with deionised water. One dispersible tablet was dissolved in 1000 mL of deionised water. 

Ten tablets of the MPG-containing drug Captimer (MIT Gesundheit GmbH, Germany) were weighed and pulverised. A powder quantity equivalent to 100 mg of MPG was dissolved in 300 mL of deionised water, filtered through filter paper (Blue ribbon, S&S, Germany), and the filtrate collected in a 500 mL volumetric flask was diluted by deionised water to the nominal volume. It is noteworthy that such solutions are not stable and should be analysed within 24 hours. These solutions were further diluted quantitatively with water to obtain suitable concentrations for the analysis by the proposed spectrophotometric method.

### 2.3. Procedures

Acetate buffer (20.0 mL, pH 3.6) was pipetted into a 25.0 mL calibrated flask. Then 1.25 mL of 10.0 mM Fe(III), 1.25 mL of 10.0 mM TPTZ, and 1.0 mL of NAC or MPG solutions were added. The flask with reaction solution was filled to the nominal volume with deionised water, mixed well, and kept at room temperature (about 25°C) for 30 min (MPG) or 60 min (NAC). The absorbance of the produced Fe(II)-TPTZ complex was measured at *λ* = 593 nm against a blank solution, prepared in the same manner with 1.0 mL water instead of 1.0 mL sample solution. The absorbance of the obtained complex remains constant for at least 24 hours. NAC and MPG concentrations in pharmaceutical preparations were determined by using daily prepared calibration curves. The eleven solution of every analyte were prepared for the concentration range from 1.0 *μ*M to 100.0 *μ*M. The standard solutions were prepared by appropriate serial dilution from the stock solutions.

## 3. Results and Discussion

The proposed method is based on the coupled redox-complexation reaction. In the first (redox) step of the reaction (see (1)), RSH compound (NAC or MPG) reduces Fe(III) to Fe(II) whereas RSH molecules themselves oxidize to thiyl radicals RS^●^ which combine to form the disulfide RSSR. In the second step of the reaction (see (2)), *in situ* formed Fe(II) is immediately complexed by 2 molecules of TPTZ to form the deep-blue coloured, highly stable Fe(TPTZ)_2_^2+^ complex which absorbs light at *λ*_max_ at 593 nm. The net overall reaction can be expressed by reaction (3):

(1) $$\begin{array}{c} {\text{Fe}}^{3+}+\text{RSH}⇄{\text{Fe}}^{2+}+{\text{H}}^{+}+{\text{RS}}^{●} \end{array}$$

(2) $$\begin{array}{c} {\text{Fe}}^{2+}+2\;\text{TPTZ}⇄{\text{Fe}{\left(\text{TPTZ}\right)}_{2}}^{2+} \end{array}$$

(3) $$\begin{array}{c} 2\;{\text{Fe}}^{3+}+2\;\text{RSH}+4\;\text{TPTZ}⇄2\;{\text{Fe}{\left(\text{TPTZ}\right)}_{2}}^{2+}+\text{RSSR}+2\;{\text{H}}^{+} \end{array}$$

Krishnamurti and Huang have reported that the complexation of TPTZ is specific for Fe(II) so that this reaction can be performed in the presence of large amounts of Fe(III) [27]. The results of the present study confirm these previous results for the drugs NAC and MPG serving as the reducing agents. 

In the literature, we were not able to find the standard reduction potential for NAC and MPG. However, the calculated formal potential of the Fe(III)/Fe(II) couple of 0.578 V, equations (4) and (5) indicate that its oxidizing power in solution with TPTZ is more negative than in solution with 1,10-phenanthroline (1.197 V). This means that the proposed method with the TPTZ is selective for the determination of NAC and MPG. Thiols or other reducing substances with standard (formal) potentials higher than 0.6 V would not interfere in the proposed method (see (6));

(4) $$E_{1}^{0\prime }=E_{\text{F}{\text{e}}^{3+}/\text{F}{\text{e}}^{2+}}^{0}-\frac{0.0592}{2}\cdot \log\;{\left(\frac{\alpha_{\text{F}{\text{e}}^{2+}}}{\alpha_{\text{F}{\text{e}}^{3+}}\cdot {\left[\text{TPTZ}\right]}^{2}}\right)}^{2},$$

(5) $$E_{1}^{0\prime }=0.771\;\text{V}-0.0592\cdot \log\;\frac{1.265\times 10^{-5}}{0.077\cdot {\left(3\times 10^{-4}\right)}^{2}}=0.578\;\text{V}$$

(6) $$E_{2}^{0\prime }=E_{\text{RSSR}/\text{RSH}}^{0}-0.0592\cdot \text{pH}.$$

In previous work, we found that the initial redox-complexation reaction rate is higher with MPG compared to NAC [28, 29]. In the coupled redox-complexation reaction with MPG, the steady state value of the absorbance is reached after 30 min while in the reaction with NAC the steady state value of the absorbance is reached after 60 min. With both thiols, maximum absorbance remains stable for at least 24 hours. This observation is of particular importance in quantitative analyses. 

### 3.1. Effect of pH

**Figure 1:**
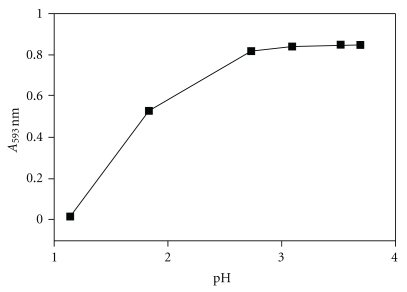
Effect of the pH value of the reaction mixture on the absorbance of the Fe(TPTZ)_2_^2+^ complex. For each pH value, the absorbance was recorded 30 minutes after beginning of the reaction. Triplicate measurements, practically the same, were done for each pH value. Initial concentrations were 40 *μ*M for MPG, 0.2 mM for Fe(III), and 0.2 mM for TPTZ. Reaction temperature was 25°C.

Equation (6) indicates that the potential for the redox system RSH/RSSR depends upon the pH value of the reaction mixture. The effect of the pH was therefore investigated over the range 1.0–4.0 using 0.1 M HCl for pH 1, 0.01 M HCl for pH 2, and acetate buffer for the pH values 3.2, 3.5 and 3.6. In this experiment MPG was used as the reducing agent in the coupled redox-complexation reaction. The results are shown in Figure 1. 

The absorbance at 593 nm increased with increasing pH up to the value of 3.6. However, precipitation of iron hydroxide occurred at the pH above 3.8. Therefore, a buffered reaction medium of pH 3.6 was chosen as a compromise for keeping Fe(III) in solution and achieving quantitative formation of the Fe(TPTZ)_2_^2+^ complex which is stable in the pH range 3.4–5.8 [26].

### 3.2. Effect of the Concentration of Fe(III) and TPTZ

**Figure 2:**
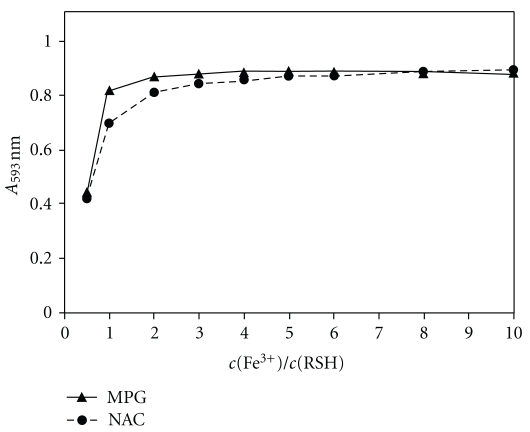
Effect of the Fe(III) concentration on the absorbance at 593 nm for the fixed concentration of 40 *μ*M for the thiol (RSH) compound, that is, NAC or MPG, at pH 3.6 and 25°C. The concentration of TPTZ was 0.2 mM.

The influence of the Fe(III) concentration on the determination of NAC and MPG at the fixed concentration of 40 *μ*M each was studied in the concentration range from 20 *μ*M to 400 *μ*M, allowing a molar ratio Fe(III)/RSH from 0.5 to 10. In Figure 2, the absorbance measured at 593 nm is plotted versus the molar Fe(III)/RSH ratio for NAC and MPG. Figure 2 shows that the reaction can be forced to completion by increasing the Fe(III)/RSH ratio, for instance by increasing the Fe(III) concentration. 

**Figure 3:**
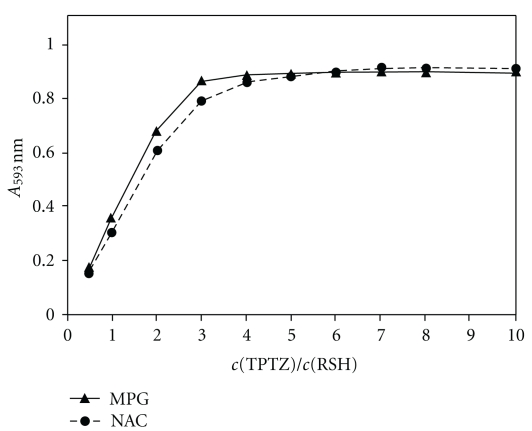
Effect of the TPTZ concentration on the absorbance at 593 nm for the fixed concentration of 40 *μ*M of the thiol (RSH) compound, that is, NAC or MPG, at pH 3.6 and 25°C. The concentration of Fe(III) was 0.2 mM.

The influence of the TPTZ concentration on the analysis of NAC and MPG at the fixed concentration of 40 *μ*M RSH compound was studied in the range from 20 *μ*M to 400 *μ*M allowing a TPTZ/RSH molar ratio of 0.5 to 10.  Figure 3 shows that absorbance increased with increasing TPTZ/RSH molar ratio, that is, with increasing TPTZ concentration, to reach its maximal value at a molar excess of five.

### 3.3. Effect of the Temperature

The effect of the reaction temperature on the signal intensity was examined by varying the temperature from 25°C to 40°C using the thermostated water pump. We found that the reaction rate increased by elevating reaction temperature (see [28, 29]). Since the proposed method is an equilibrium method, signal is recorded when the reaction reaches the state of equilibrium. The signal intensity in the state of equilibrium is the same for all the examined temperatures. However, for practical reasons the ambient laboratory temperature of 25°C was finally used.

### 3.4. Analytical Characteristics

The linearity of the method was investigated under the optimized conditions for NAC and MPG in the concentration range from 1.0 to 100.0 *μ*M. Straight lines were obtained from linear regression analysis of the absorbance at 593 nm and the drug concentration (Table 1). Expectedly, very similar results were obtained for NAC and MPG. The lowest quantifiable concentration of NAC and MPG by this method was 1.0 *μ*M each.

### 3.5. Interferences Studies

The effect of some possible interfering cations and anions on the analysis of a fixed concentration of 40.0 *μ*M for NAC and MPG was investigated for the maximum molar ratio of foreign ions. The influence of excipients that can commonly accompany NAC and MPG in pharmaceutical formulations was also studied. 

The tolerance is defined as the foreign-ion/excipient concentration causing an error smaller than ±5% for the determination of the analyte of interest. The tolerable concentration of KNO_3_ and Na_2_SO_4_ was 40.0 mM (molar ratio, 1000 : 1). The tolerable concentration of glucose, fructose, sucrose, boric acid, and acetic acid was 20.0 mM (molar ratio, 500 : 1). Thus, the commonly excipients glucose, fructose, and sucrose do not interfere with the analysis of NAC and MPG because they essentially do not react with the oxidizing agents. It should be emphasized that the contaminant/analyte concentration ratios studied in the present work are much higher than those normally found in commercial pharmaceutical products.

The tolerable concentration of some other thiols, that is, D-penicillamine, L-glutathione, and L-cysteine, was 40.0 *μ*M (molar ratio, 1 : 1). These experiments confirmed above-mentioned theoretical consideration. Thiols or other reducing substances with standard (formal) potentials higher than 0.6 V would not interfere in the proposed method.

### 3.6. Application of the Method

In order to evaluate the potential of the proposed method to the analysis of real samples, the method was applied to three pharmaceutical formulations of the drugs NAC and MPG. The results of these analyses are presented in Tables 2 and 3. 

For comparison, the spectrophotometric method reported by Raggi et al. [24] was used for the parallel assay of the same batch tablets. These authors used 1,10-phenantroline as an chromogenic reagent, instead of TPTZ which is used in the present work. As shown in Table 2, there were no significant differences between the values obtained by the reported method [24] and those obtained by the proposed method (*P* > 0.1, Student *t*-test). This actually suggests that the proposed method is accurate and precise as the earlier reported method [24].

The accuracy of the method was further ascertained through the recovery studies. To the drug solutions of the granules or the tablet powder, the standard solutions of the synthetic NAC or MPG were added at four different concentrations. The total content was determined by the proposed method. The recovery of added NAC or MPG was 98–102% (Table 3). 

These results indicate that the proposed method is accurate for the determination of NAC and MPG in their commercially available pharmaceutical preparations without any significant interference by common pharmaceutical excipients which do not absorb light in the visible region. 

Performance characteristic of existing equilibrium spectrophotometric methods [19, 21–24] and the proposed method are compared in Table 4.

The proposed method is free from drastic experimental conditions such as heating unlike some of reported methods. Some other thiol compounds do not interfere in the proposed method at molar ratio 1 : 1. It is also worth mentioning that the proposed method was performed in the visible region (*λ* = 593 nm) away from the UV-absorbance of the UV-absorbing interfering excipient materials, which might be dissolved from pharmaceutical formulations.

## 4. Conclusion

Time, cost, and efficiency are essential considerations in pharmaceutical industry. Undoubtedly, HPLC is one of the most widely used techniques in routine analysis of pharmaceuticals, but it involves expensive instrumental set which many laboratories in developing and underdeveloped countries can not afford. The proposed equilibrium spectrophotometric method based on the coupled redox-complexation reaction of the thiol drug NAC or MPG with Fe(III) and TPTZ can be applied in every analytical laboratory as a reliable method for the determination of NAC or MPG in pharmaceutical preparations. Due to the use of TPTZ fast colour development reaction is easily conducted at the room temperature. The coloured Fe(TPTZ)_2_^2+^ complex is stable in an extended period of time up to 24 hours. Regular excipients and additives present in the pharmaceutical preparations of NAC and MPG do not interfere in this method.

## Figures and Tables

**Table 1:** Spectral characteristics and analytical parameters of the method for NAC and MPG under optimum reaction conditions.

Analytical parameter	NAC	MPG
*λ*_max_ (nm)	593	593
**ε** (M^−1^ cm^−1^)^(a)^	2.2 × 10^4^	2.2 × 10^4^
Sandell's sensitivity (*μ*g cm^−2^)^(a)^	7.5 × 10^−3^	7.6 × 10^−3^
*m* (slope) ± SD	2.14 × 10^4^ ± 0.0077	2.15 × 10^4^ ± 0.0074
*z* (intercept) ± SD	0.0025 ± 0.0026	0.0036 ± 0.0033
Linear regression coefficient (*R* ^2^)	0.9999	0.9999
Beer's law range (*μ*M)	1.0 to 100.0	1.0 to 100.0
Number of points/replicates	11/3	11/3
Detection limit (*μ*M)^(b)^	0.14	0.13
Quantitation limit (*μ*M)^(c)^	1.0	1.0

^(a)^Average of eleven determinations.

^(b)^Detection limit = 3 s_b_/m (three standard deviations for a blank divided by the slope of the calibration curve).

^(c)^Quantitation limit = at least 10 s_b_/m (ten standard deviations for a blank divided by the slope of the calibration curve).

**Table 2:** Determination of NAC and MPG in their pharmaceutical formulations by the proposed spectrophotometric equilibrium method and by a literature method [24].

Pharmaceutical preparation	Present work^(a)^ mg	Method from [24] ^(a)^ mg
Fluimukan^(b)^ (NAC)	202.0 ± 1.9	202.9 ± 3.2
Fluimukan Akut^(c)^ (NAC)	605.9 ± 6.1	606.9 ± 7.2
Captimer^(d)^(MPG)	99.2 ± 0.8	99.0 ± 1.0

^(a)^Average of three determinations ± SD.

^(b)^Granules containing 200 mg NAC and excipients.

^(c)^Dispersible tablets containing 600 mg NAC and excipients.

^(d)^Tablets containing 100 mg MPG and excipients.

**Table 3:** Accuracy (recovery, %) of the proposed method for the determination of NAC and MPG in two pharmaceutical formulations.

Sample	Added *μ*g mL^−1^	Found^(a)^ *μ*g mL^−1^	Recovery %
Fluimukan (NAC)	0.0	200.1 ± 0.4	Not applicable
	50.0	249.1 ± 1.6	98.2
	100.0	301.2 ± 1.8	101.2
	150.0	348.2 ± 2.1	98.8
	200.0	404.4 ± 2.2	102.2

Captimer (MPG)	0.0	100.2 ± 0.6	Not applicable
	50.0	149.3 ± 1.1	98.6
	100.0	198.4 ± 1.3	98.4
	150.0	252.5 ± 2.4	101.7
	200.0	303.9 ± 3.1	101.9

^(a)^Average of three determinations ± SD.

**Table 4:** Comparison of the equilibrium spectrophotometric methods for NAC and MPG determination.

Analyte	Reagent(s) used	*λ*_max_ (nm)	Beer's law range (*μ*M)	**ε** (M^−1^ cm^−1^)	Reference
NAC, MPG, penicillamine	Fe(III)/1,10-phenanthroline	515	4.0–80.0	1.1 × 10^4^	[24]
NAC	PdCl_2_	375	24.5–400.0	Not reported	[23]
NAC	o-phthalaldehyde/isoleucine	335	3.0–300.0	6.3 × 10^4^	[22]
Cysteine, NAC	Fe(III)/ferrozine	562	0.1–36.8	2.3 × 10^4^	[21]
NAC	IO_3_^−^/leucoxylenecyanol	613	1.2–9.8	9.6 × 10^4^	[19]
NAC MPG	Fe(III)/TPTZ	593	1.0–100.0	2.2 × 10^4^	Present work
